# SPoRE: a mathematical model to predict double strand breaks and axis protein sites in meiosis

**DOI:** 10.1186/s12859-014-0391-1

**Published:** 2014-12-11

**Authors:** Raphaël Champeimont, Alessandra Carbone

**Affiliations:** Sorbonne Universités, UPMC Univ Paris 06, UMR 7238, Laboratoire de Biologie Computationnelle et Quantitative, Paris, F-75006 France; CNRS, UMR 7238, Laboratoire de Biologie Computationnelle et Quantitative, Paris, F-75006 France; Institut Universitaire de France, Paris, 75005 France

**Keywords:** Recombination, 3D chromosomal structure, Genome, Gene, Modeling, Intergenic region, *Saccharomyces cerevisiae*

## Abstract

**Background:**

Meiotic recombination between homologous chromosomes provides natural combinations of genetic variations and is a main driving force of evolution. It is initiated via programmed DNA double-strand breaks (DSB) and involves a specific axial chromosomal structure. So far, recombination regions have been mainly determined by experiments, both expensive and time-consuming.

**Results:**

SPoRE is a mathematical model that describes the non-uniform localisation of DSB and axis proteins sites, and distinguishes high versus low protein density. It is based on a combination of genomic signals, based on what is known from wet-lab experiments, whose contribution is precisely quantified. It models axis proteins accumulation at gene 5’-ends with a discrete approximation of their diffusion and convection along genes. It models DSB accumulation at approximated gene promoter positions with intergenic region length and GC-content. SPoRE can be used for prediction and it is parameterised in an obvious way that makes it easy to understand from a biological viewpoint.

**Conclusions:**

When compared to *Saccharomyces cerevisiae* experimental data, SPoRE predicts axis protein and DSB positions with high sensitivity and precision, axis protein density with an average local correlation *r*=0.63 and DSB density with an average local correlation *r*=0.62. SPoRE outbreaks previous DSB predictors, which are based on nucleotide patterning, and it reaches 85% of success rate in DSB prediction compared to 54% obtained by available tools on a benchmarked dataset.

SPoRE is available at the address http://www.lcqb.upmc.fr/SPoRE/.

**Electronic supplementary material:**

The online version of this article (doi:10.1186/s12859-014-0391-1) contains supplementary material, which is available to authorized users.

## Background

In sexually reproducing eukaryotes, the production of gametes relies on meiosis, during which a diploid cell is divided into four haploid cells. A critical step is homologous recombination between homologous chromosomes, in which both crossover and non-crossover events occur, resulting in a different gene content of the offspring chromosomes. This allows evolution to explore different allelic combinations through recombinations that are more likely to occur in some regions than others [1,2] and that are initiated by DNA double-strand breaks (DSBs) [3].

The Spo11 protein, a transesterase highly conserved through evolution [4] and required for meiotic recombination in *Saccharomyces cerevisiae* [3], *Caenorhabditis elegans* [5], Drosophila [6] and mammals [7], causes DSBs. Two Spo11 proteins work in concert to cut both DNA strands and, after the cleavage, each Spo11 is bound to a DNA fragment [8]. This property has been used to make a high-resolution DSB density map of the *S. cerevisiae* genome [9] revealing that DSBs are more abundant before gene starts [9]. It is also known that DSB frequency is strongly correlated with GC-content [10,11], open chromatin structure [12-16] and histone methylation [17,18].

A specific chromosomal structure is formed during meiosis, and plays a key role in recombination events. The formation of this structure is due to bonding of cohesin and several other proteins on specific DNA sites, and to their assembly in a protein complex forming an axis [19-21]. The DNA lying outside protein binding sites forms loops along the axis [11,22,23]. Axes of homologous chromosomes are themselves bound to each other by transversal filaments made of (Zip1 in *S. cerevisiae*, Sycp1 in mammals) proteins, forming the so-called synaptonemal complex.

In *S. cerevisiae*, the structural axis is formed by cohesin (Rec8), Red1 and Hop1 proteins [19,20]. It has been shown that proteins Mer2, Rec114, and Mei4 bind to DNA at axis sites, rather than to loop sequences, and that their association depends on Red1 and Hop1. In turn, Red1 deposition depends on Rec8 cohesin in certain regions, but not in others, and in both cases, their local distribution is very similar [24]. The global correlations are *r*=0.88 between Red1 and Hop1, *r*=0.57 between Red1 and Rec8, and *r*=0.38 between Hop1 and Rec8. Also, cohesin (and Red1) density is higher in *convergent regions*, that is intergenic regions characterized by two gene ends, and is correlated with AT-content [25], but no specific cohesin-DNA binding motif has been identified. Red1 density is locally negatively correlated with DSB density (see Figure two E in [24]).

Many questions remain open on the chromosomal axis formation. Can we model the accumulation of axial proteins and DSBs on the chromosomes entirely from genomic factors? If so, what is the contribution made by each one of them?

A number of genomic markers (like gene start, gene end, GC-richness) associated to high-density sites have been highlighted by experiments [10,26-29], but, once combined together, how well do they explain the frequency of occurrence of proteins or DSBs in a given site along the axis? In other words, can we create a mathematical model, based on genomic markers, and a tool that can predict axial proteins and DSB? With a rapid increase of sequenced genomes, it is highly desirable to develop automated methods for timely identifying recombination hotspots.

Computational predictions of recombination hotspots have been based on nucleotide sequence content. These approaches take into account, more [30] or less [31,32] explicitly, sequence order effects. But the accuracy of these algorithms still needs further improvement and more formal mathematical approaches, considering mechanisms of chromatin remodeling during meiosis, could be a possible way to proceed.

Here, we provide a mathematical framework, called SPoRE for “SPots of REcombination”, that allows us to model axis proteins and DSB localization and density along chromosomes. SPoRE models axis proteins and DSB distributions based on genomic information. For axis proteins, SPoRE uses gene stop codon positions and gene lengths as its only input, while for DSBs, it uses the order of the genes defining intergenic regions, intergenic region lengths and GC-richness. Based on these genomic markers, SPoRE models *S. cerevisiae* experimental data for Red1 and Spo11 distributions [9,24] accurately. We used SPoRE to make predictions on three more yeast species, *Lachancea kluyveri*, *Kluyveromyces lactis* and *Schizosaccharomyces pombe*. Finally, we compared it to available tools predicting DSB hotspots and coldspots, and demonstrated its higher performance.

## Results and discussion

### The model and the algorithm

**Figure 1:**
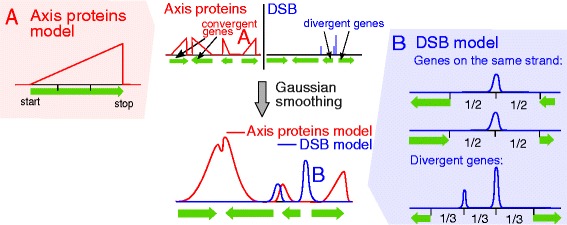
**Summary of SPoRE modeling approach.** Two main steps constitute the approach and they are described from top to bottom (center). First, SPoRE considers a set of positions to which it assigns weights. Axis proteins (red) and DSBs (blue) involve convergent genes and divergent genes, respectively. In the drawing, locations with non-zero weight are indicated by colored vertical bars (height represents importance) and triangles: convergent genes for axis proteins and divergent regions for DSBs display the highest weights (top). Then, SPoRE smooths the distribution of weights with a Gaussian kernel (bottom) modeling, in this way, the diffusion of the proteins around their main sites. The red box on the left (**A**; axis proteins) and the blue box on the right (**B**; DSB) describe some details of SPoRE models.

SPoRE modeling of axis proteins and DSBs relies on a general principle that can be summarized in two main steps (Figure 1). First, it defines a set of positions on the genome where proteins might accumulate, and sets a weight for each of these positions according to gene annotation. This weight is used as an indicator of the density of the proteins. In the second step, it makes a smooth curve using a Gaussian kernel of the distribution of weights along the genome.

**Figure 2:**
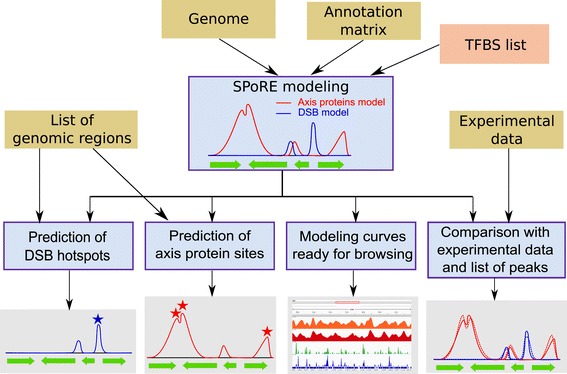
**SPoRE flowchart.** SPoRE takes several input files (brown boxes); the input in the orange box is optional. SPoRE implements the construction of the modeling curves for axis proteins and DSBs, as described in Figure 1 (blue box, top), and uses these curves as input for 4 algorithmic tasks (bottom blue boxes; outputs in grey boxes): 1. The prediction of DSB hotspots. Starting from a list of genomic regions, it decides whether these regions are susceptible to DSB or not; 2. The prediction of axis proteins sites. As in 1, it makes predictions starting from a list of genomic regions provided by the user; 3. The production of ready for browsing output files describing the axis proteins and the DSB modelling curves (see Additional file 1: Figure S1); 4. The comparison of SPoRE models (solid line) to experimental data (dashed lines).

This main computational core in SPoRE, takes as input a genome and its gene annotation, and provides as output the modeling curves describing DSBs and axis proteins distribution along the whole genome (Figure 2). A list of Transcription Factor Binding Sites (TFBS) can be provided as input for more accurate promoter region detection. This intermediate output is used by SPoRE to provide four kinds of data: 1. It produces the curves modeling the density of DSBs and axis proteins along the whole genome, in a format that is ready for browsing (see Additional file 1: Figure S1). 2. Given a list of intervals on the genome, it predicts whether they are hot or cold spots for DSBs. 3. Given a list of intervals on the genome, it predicts whether they are axis sites. 4. Given an experimental curve defined over the genome, it compares the DSB and axis proteins model curves with experimental data and provides Pearson and Spearman local correlation coefficients between them. Also, it compares the peaks of the model curves with the peaks of the experimental curve, computing PPV and sensitivity.

SPoRE can be easily used. It takes as input a genome and its associated gene annotation, and all its parameters are automatically computed on the input genome. Also, SPoRE works on scaffolds, not only on fully assembled chromosomes, since its minimal requirement is ORF annotation.

#### Analysis of convergent and divergent regions

Our intuition on the positioning of high-density hotspots for axis proteins and DSBs was developed with the analysis of the *S. cerevisiae* experimental data in [9,24]. In understanding these data, we focused on convergent and divergent regions, instead of considering the start and the end of genes as previously done (compare Additional file 1: Figure S2 to Additional file 1: Figure S3). The plots, reported in Additional file 1: Figure S2A-F, highlight characteristics of the data when displayed for convergent and divergent intergenic regions. Notice that in [25], it was already observed that meiotic cohesin preferably accumulates in convergent regions (Additional file 1: Figure S2A), with an extreme bias against regions in which transcription is diverging (Additional file 1: Figure S2B).

By focusing at convergent and divergent regions, we observe (and provide with that a precise numerical evaluation) that:

1. The local negative correlation between Red1 and DSBs localizations observed in [24,25] physically corresponds to convergent and divergent regions, where convergent regions present high average Red1 density and almost no presence of DSBs (Additional file 1: Figure S2A and D), while divergent regions present a high average Spo11 density and an important decrease in Red1 (Additional file 1: Figure S2B and E);

2. Red1 density is much higher at gene 3’-ends than it is at gene 5’-ends, and yet even higher when we consider only convergent intergenic regions, having two gene 3’-ends (Additional file 1: Figure S2A and C);

3. DSB density is twice as high in divergent regions, having two gene starts, than in *tandem regions*, that is intergenic regions between co-directional genes (Additional file 1: Figure S2E-F);

4. DSB peaks are localized in promoter regions. This observation has already been made long ago [28], and was confirmed with the high-resolution DSB density in [9], in which the authors found that 88.2% of DSBs overlap with promoters. This can be seen in the DSB distribution in large divergent regions (Additional file 1: Figure S4C-D). For the vast majority of intergenic regions (of <800*nt* in length), the DSB peaks appear roughly centered in the middle of divergent regions (Additional file 1: Figure S2E-F), this position well approximating promoter locations.

From [24], we also observed that:

5. The shape of the distribution of Red1 proteins along genes (Additional file 1: Figure S5A), highlights a linear increase of the amount of Red1 proteins towards the gene end. On single genes this increasing distribution is not sharply distinguishable but when considering all genes together, it becomes gradually more pronounced in longer genes. In particular, the area under the distribution curves increases proportionally to gene length.

6. The distributions of Rec8 and Hop1 in intergenic regions have a shape similar to the Red1 distribution (see Additional file 1: Figure S10).

#### Axis proteins model

In a first attempt, axis proteins could be modeled by using gene 3’-ends as reference positions and by associating to each position a weight corresponding to the length of the relative gene. This simple model implies that convergent regions are governed by weights defined as the “sum” of two gene lengths, that tandem regions are modeled by the length of only one gene, and that divergent regions are ignored. It captures well some characteristics observed in *S. cerevisiae* experimental data: convergent regions host about the double amount of Red1 compared to tandem regions, when we subtract the base noise level (see Additional file 1: Figure S2A and Additional file 1: Figure S2C) and the amount of Red1 at gene 3’-ends augments with gene length (Additional file 1: Figure S4A).

SPoRE is based on this simple model but it also describes, in an explicit way, the spread of Red1 proteins along the gene. This Red1 spreading is likely due to two processes, one of diffusion and one of convection of proteins. Since experimental measures of diffusion constants produced highly varying values depending on the organism and on the protein [33], and that measures of convection constants are also organism and gene dependent [34], we cannot directly use them to model the curves in Additional file 1: Figure S5A. Then, we discretely approximated the curves through a linearly increasing curve that begins at the start of the gene and increases to its maximum value at the gene end, as in Additional file 1: Figure S5C. Since we wish the amount of axis proteins per gene to be proportional to gene length, we set the “triangle” height to be the same for all genes. As a consequence, the area of the triangle is proportional to gene length, as described by experimental data (Additional file 1: Figure S5A).

The precise mathematical formulation of SPoRE model is the following. First we define the raw curve before smoothing:

$$h(x) = \sum\limits_{g\in G} \textbf{1}_{[a_{g},b_{g}]}(x).\frac{x-a_{g}}{b_{g}-a_{g}} $$

where *G* is the set of all genes and *x* the position (in nucleotides) on the genome, *a*_*g*_ is the position of the start codon of *g*, and *b*_*g*_ is the position of its stop codon. The function **1**_[*a*,*b*]_(*x*) has value 1 if *x*∈ [ *a*,*b*] and 0 otherwise.

Then we apply a kernel-based smoothing with a Gaussian kernel to *h*(*x*). Namely, we compute the convolution with a Gaussian kernel *K* to obtain the final function *f*_Red1_ which is our Red1 model curve:

$$f_{\text{Red1}}(x) = (h*K)(x) = \int_{-\infty}^{+\infty} h(x) \cdot e^{-\frac{(t-x)^{2}}{2\sigma_{\text{smooth}}^{2}}}. dt $$

where *σ*_smooth_ is 1500 nucleotides.

#### DSB model

SPoRE localizes DSBs in promoter regions. Since these regions are not easily identifiable, SPoRE follows a few rules to approximate their position in an intergenic region: 1. if the region is convergent, then no DSB is supposed to occur in it, 2. if the region is between two co-oriented genes (tandem region), then DSBs are located at the center of the intergenic region, accounting for the promoter of the starting gene, 3. if the region is divergent, then DSBs are located at two positions, at 1/3 and at 2/3 of the intergenic region respectively, corresponding to the two promoters. In cases 2 and 3, the amount of DSBs is also modeled to be dependent on the average GC-content within a window (see Methods). If TFBS are available, SPoRE can use them to identify the promoter region of a gene and replace the location identified by steps 2 and 3 above with a more accurate evaluation of the promoter location.

SPoRE adds one more contributing factor to the above model: the intergenic region length. For this, it makes sure that the contribution of very long intergenic regions would not be penalized by high weights, and fixes a maximum weight threshold to a value IRL_max_.

Formally, SPoRE modeling curve *f*_DSB_(*x*) is defined as:

$$ \sum\limits_{g\in G} \min(\text{irl}_{g}, \text{IRL}_{\max}) \cdot (\max(0, \text{gc}(p_{g}) - \text{GC}_{\min}))^{2} \cdot e^{-\frac{(x-p_{g})^{2}}{2\sigma_{\text{smooth}}^{2}}} $$

where *G* is the set of all genes, *x* the position (in nucleotides) on the genome, irl_*g*_ is the intergenic region length before the gene (on the strand where *g* is lying). The position *p*_*g*_ depends on both the orientation of *g* and the position of gene *g*^′^ preceding *g*; gc(*p*_*g*_) is the smoothed GC content at position *p*_*g*_. Let [*a*,*b*] be the intergenic region and *a* be the start codon position of *g*, then:

$$ p_{g}\! =\! \left\{ \begin{array}{l l} a + (b-a)/2 & \,\,if\,\, \textit{g}\,\, and\,\, g^{\prime}\,\, are\,\, on\,\, the\,\, same\,\, strand\\ a + (b-a)/3 & \,\,if\,\, \textit{g}\,\, and\,\, g^{\prime}\,\, are\,\, on\,\, opposite\,\, strands \end{array} \right. $$

The two thresholds IRL_max_ and GC_min_ are defined as IRL_max_=*μ*_IRL_+*σ*_IRL_ and GC_min_=*μ*_GC_−3*σ*_GC_, where *μ*_IRL_ (*μ*_GC_) and *σ*_IRL_ (*σ*_GC_) are mean and standard deviation of the distribution of intergenic region lengths (*GC* content) over the whole genome. The quadratic term describes a preferred DSB concentration in regions with a higher GC content.

This model takes into account the observation that divergent regions host about the double amount of DSBs compared to tandem regions (indeed, 2 gene starts instead of 1 in an intergenic region influence twice as much the average DSB density) and that, at large scale, on the thousands of base pairs scale, GC-content correlates with DSBs [10].

### Comparison with experimental data

SPoRE has been constructed to predict DSB and axis proteins distribution along chromosomes, and to measure the importance of different factors in this prediction. To evaluate how accurate SPoRE modeling is, we performed four types of analysis:

*a*. experimental data on Red1 [9] and Spo11 [24] proteins obtained for the *S. cerevisiae* genome were considered and the local/global Pearson and Spearman correlations between SPoRE modeling curves and experimental curves were computed. The distribution of peaks, characterizing sites of highest protein concentration, along the two curves was studied. Several models, characterized by different combinations of genomic signals, were tested to numerically evaluate the impact of each signal.

*b*. Coherence of SPoRE predictions was tested on two experimental datasets [24,35] related to axis proteins and DSBs.

*c*. SPoRE was run on four yeast species.

*d*. SPoRE was compared to existing DSB predictors, all based on machine learning [30-32].

#### SPoRE model and axis proteins in *S. cerevisiae*

SPoRE model (that is model 3 in Table 1) is based on the hypothesis that axis proteins accumulate at the end of genes, that genic region length is the main factor for protein density, and that taking into account protein diffusion and convection along the gene improves precision. SPoRE reaches average Pearson local (global) correlation *r*=0.63 (*r*=0.54; Additional file 1: Figure S7A) and Spearman’s local (global) correlation *ρ*=0.63 (*ρ*=0.60). We note that lower correlations are obtained when an increasing distribution of proteins along the gene is omitted (model 2 in Table 1): Pearson’s local (global) correlation is *r*=0.58 (*r*=0.52), and Spearman’s local (global) correlation is *ρ*=0.54 (*ρ*=0.51).

**Table 1:** **Performance of SPoRE and other models for axis proteins and for DSBs**

**Axis proteins - Red1**						
**Model description**			**Pearson**		**Spearman**	
			**correlation**		**correlation**	
**Id**	**Positions**	**Weights**	**Loc**	**Glo**	**Loc**	**Glo**
1	Gene ends	1	0.14	0.11	0.13	0.11
2	Gene ends	Gene length	0.58	0.52	0.54	0.51
3	Diffusion along gene	Gene length	**0.63**	**0.54**	**0.63**	**0.60**
**DSB - Spo1**						
**Model description**			**Pearson**		**Spearman**	
			**correlation**		**correlation**	
**Id**	**Positions**	**Weights**	**Loc**	**Glo**	**Loc**	**Glo**
1	Gene starts	1	0.34	0.28	0.68	0.65
2	Gene starts	Gene length	0.26	0.21	0.65	0.63
3	Promoters	1	0.48	0.40	0.74	0.71
4	Promoters	*IRL*	0.50	0.41	0.74	0.70
5	Promoters	*GC*	0.58	0.52	0.75	**0.72**
6	Promoters	*G* *C*×*I* *R* *L*	**0.62**	**0.56**	**0.76**	**0.72**

Local and global Pearson and Spearman correlation coefficients have been calculated between different model curves and *S. cerevisiae* experimental data for axis proteins [9] and DSBs [24]. Bold characters highlight best performance. Different models are characterized by different weighting factors (column “weights”). For DSB analysis, *GC* is GC-content smoothed with a Gaussian kernel of 1000 nucleotides; *IRL* is the intergenic region length, or ***IRL***_***max***_ if the region is too large (see Methods). SPoRE model for axis proteins is number 3, and for DSBs is number 6. Values are output of the SPoRE program (Figure 2, bottom right). See also the correlation curves for models 3 and 6 in Additional file 1: Figure S7. All p-values associated to both Pearson and Spearman global correlations are lower than ***10e*** ^***−15***^ (even for weak correlations such as 0.11). Highest correlations are highlighted in boldface.

**Figure 3:**
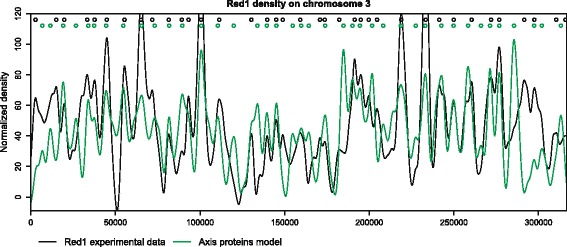
**SPoRE model for axis proteins compared to experimental data in** ***S. cerevisiae*** **chromosome 3.** Red1 density curve [24] (black) and SPoRE axis proteins modelling curve (green) on chromosome 3. Colored circles on the top of the plot mark peaks of the curves.

**Figure 4:**
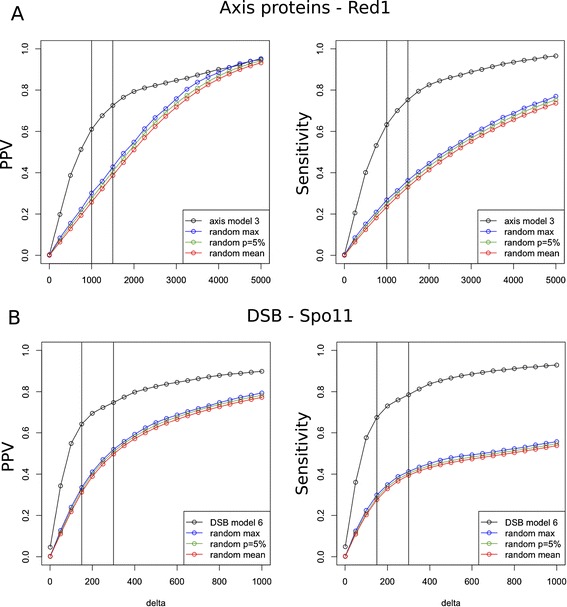
**SPoRE performance in detecting axis proteins and DSB hotspots for** ***S. cerevisiae*** **.** Peaks localisation (not density) in SPoRE curves is compared to peaks localisation in experimental curves for axis proteins [9] **(A)** and DSBs [24] **(B)**. Positive Predictive Value (PPV) and Sensitivity (see Methods) obtained with SPoRE models (number 3 for axis proteins and number 6 for DSBs) are reported for increasing values of the parameter *Δ*, representing the maximum distance allowed between two peaks to say that they match. The vertical bars in the plots correspond to *Δ*=1 kb and 1.5 kb in **A** and to *Δ*=150 nt and 300 nt in **B**. Different random models are used to analyze SPoRE behavior (see Methods): best PPV/sensitivity over 1000 simulations (blue), PPV/sensitivity for a p-value of 5% (green), average PPV/sensitivity over 1000 simulations.

Red1 localization is well predicted by the position of the peaks of SPoRE modeling curve (Figure 3). For instance, along all chromosomes, 62% of real peaks are found by our model at a distance of at most *Δ*=1 kb from a predicted peak (74% at 1.5 kb), and 62% of the predicted peaks are at most 1 kb away from a real peak (73% at 1.5 kb). Sensitivity and PPV at increasing *Δ* values are illustrated by the curve plot in Figure 4A. We notice that random models, based on random selections of spots along the genome (see Methods), give much lower PPV and sensitivity values.

It is worth noticing that the usage of constant weights makes the model performance very poor, as the correlation with real data falls down to *r*=0.14 (model 1 Table 1). Strictly speaking, even the positional analysis of the peaks, as discussed above, is dependent on appropriate weight values, because a smoothing is performed before extracting the peaks (Gaussian window with *σ*=1.5 kb). Therefore, peaks result from the accumulation of high weights and they are not simply modeling gene ends. This is why model 1 (Table 1) has much lower PPV and sensitivity than model 2.

Finally, since experimental data highlight the existence of a background noise inducing a basic level of Red1 distribution along chromosomes, we verified whether, by including a fixed noise level in SPoRE model (see Methods), predictions in *S. cerevisiae* would be improving the fit or not. A minor improvement in Pearson correlation coefficients (local at *r*=0.64 and global at *r*=0.56) is observed.

#### SPoRE model and DSBs in *S. cerevisiae*

**Figure 5:**
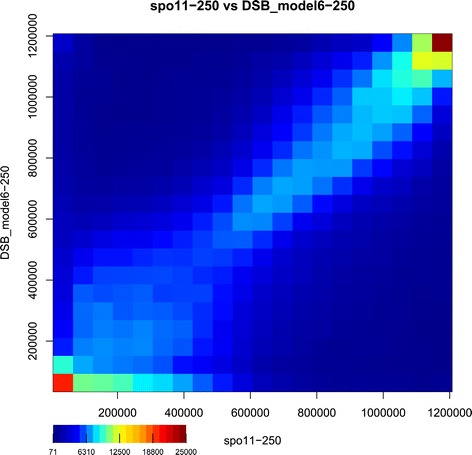
**Heatmap of the experimental Spo11 distribution curve [** 9 **] and the Spo11 SPoRE curve on the** ***S. cerevisiae*** **genome.** Pairs of *y*-values belonging to the two curves have been recorded every 10nt along the chromosomes, and a total amount of about 1.2 millions points (*y*_1_,*y*_2_) were identified, where *y*_1_ and *y*_2_ are the *y*-coordinates of the experimental and modeling curves, respectively. In the plot, the *y*-coordinates have been replaced by their ranks to allow for better visualization. The *x*-axis reports ranks from the experimental curve and the *y*-axis reports ranks from the SPoRE modeling curve. Each square in the plot describes the number of points falling into the corresponding interval of rank values. The dark red square on the top right collects picks with the highest *y*-ranks and the red square on the bottom left collects points in the experimental curve displaying no Spo11 accumulation, and therefore no DSBs.

The SPoRE model (that is model 6 in Table 1) assumes that DSBs concentrate in gene promoter positions, and that intergenic region length and GC-content are key factors for explaining DSB density. SPoRE displays a local Pearson correlation *r*=0.62 and a Spearman correlation *ρ*=0.76 with experimental data [9]. The heatmap of the experimental Spo11 distribution curve [9] and the Spo11 SPoRE modeling curve, reported in Figure 5, shows a sharp diagonal confirming the accurate prediction of the model and in particular the precise prediction of regions with high DSB density or DSB absence.

Localization of DSB high-density spots is well predicted by the position of the peaks of our modeling curve (Additional file 1: Figure S6). For instance, 64% of the predicted peaks are found at most *Δ*=150 nt away from a real peak (PPV) and 68% of the real peaks are found at less than 150 nt away from a predicted peak (sensitivity). Sensitivity and PPV at increasing *Δ* values are reported in Figure 4B. In comparison, a random model based on a random selection of spots in intergenic regions (see Methods), displays much lower PPV and sensitivity.

Although SPoRE identifies a subset of the peaks found by the model at constant weights (see sensitivity in model 3, Table 1), it clearly predicts better their heights when GC-richness and, to a lesser extent, intergenic region length are considered. The performance of these different models is reported in Table 1.

Finally, we tested whether the knowledge of TFBSs in *S. cerevisiae* [36], leading to a more accurate promoter region localization, improves SPoRE predictions or not. There is no improvement on peak heights prediction (Pearson and Spearman local and global correlation coefficients do not increase). For peak localization, PPV slightly increases to 67% and sensitivity to 69% for *Δ*=150 nt, and we conclude that a precise estimation of promoter regions helps modeling DSB localization. The effect of TFBS availability in modeling remains limited though.

#### Coherence of SPoRE predictions with two large-scale experimental datasets

SPoRE modeling curves can be used for comparison with experimental data of different origin. In this respect, we considered two different datasets.

First, as mentioned in the introduction, it has been shown previously that Red1 and Hop1 patterns are influenced by Rec8 (cohesin) patterns [24]. Hop1, for instance, is distributed almost like Red1 (local correlation is *r*=0.92, global is *r*=0.88) with which it interacts [37,38]. On the other hand, Rec8 is more abundant around centromeres than Red1/Hop1, although local variations are the same. Therefore, Rec8 global correlation with Red1 is only *r*=0.57, while its local correlation is still *r*=0.83. Because of these correlations, we expect SPoRE to be locally well correlated with Hop1 and Rec8 (data from [24]). Indeed, we find that SPoRE model has a local correlation of *r*=0.62 with Hop1 and *r*=0.60 with Rec8, compared to *r*=0.64 with Red1. This confirms that the three axial proteins share SPoRE local distribution patterns. Consistently, if we look at global correlation coefficients, SPoRE is well correlated with Hop1 (*r*=0.55) and Red1 (*r*=0.56) but weakly correlated with Rec8 (*r*=0.33).

Second, we compared SPoRE curves to histone trimethylation data. It has been observed before that H3K4 trimethylation (H3K4me3) is linked to DSBs [17]. Then, we computed correlations between H3K4me3 (data from [35]) and SPoRE modeling curve for Spo11. We find *r*=0.25, which is comparable to *r*=0.21 obtained when we correlate H3K4me3 and DSB experimental data. Similarly, with Spearman coefficients, we find *ρ*=0.61 between H3K4me3 and our model, and *ρ*=0.52 between H3K4me3 and DSB experimental data. We conclude that SPoRE model is consistent with this known interaction.

Both these examples confirm that the modeling curves are faithful approximations of experimental curves and that biological conclusions can be safely derived from them.

#### SPoRE predictions on several yeast species

The large number of sequencing projects on yeast clades and the upcoming new projects (still a few today) exploring the molecular biology of yeast species encourages the usage of predictive tools for learning about the distribution of DSB and axial proteins sites, to start comparative studies on yeasts across clades. We run SPoRE on *Lachancea kluyveri* and *Kluyveromyces lactis*. The genome of *L. kluyveri* shows a particularly high GC-content on the left-arm of the C chromosome (see Additional file 1: Figure S8) and SPoRE predicts a higher concentration of DSBs in this chromosomal arm. We note that the number of peaks within the C-left arm is comparable to other chromosomal arms, and that SPoRE detects the same number of peaks (353) than model 4, which excludes the GC factor. Namely, the GC factor in SPoRE exclusively influences DSB density and not DSB positioning, and the high number of DSBs predicted along the C-left arm is a consequence of SPoRE higher peaks rather than SPoRE higher number of peaks. Experiments in *L. kluyveri* are expected to confirm SPoRE prediction in the C-left arm of the C chromosome.

We have also run SPoRE on *Schizosaccharomyces pombe* where recombination is known to be partially dependent on DNA motifs. As expected in this species [39], SPoRE predicts a large number of DSBs in large intergenic regions. It should be noticed that in *S. pombe*, divergent and tandem regions are unusually large compared to other yeast species. In *S. cerevisiae*, *L. kluyveri* and *K. lactis* for instance, the mean length of divergent and tandem regions, is approximately 700nt while it is 1200nt for *S. pombe* (Additional file 1: Figure S9). Since SPoRE favors DSBs in tandem and divergent regions, and since the size of these regions plays an explicit role in the model, SPoRE prediction confirms the previous observations.

When comparing SPoRE predictions with the DSB distribution in *S. pombe* [40], results are much less accurate than with *S. cerevisiae*. We get a local Pearson correlation of *r*=0.36 (global correlation is *r*=0.26). Spearman correlation is better with *ρ*=0.43 (global correlation is *ρ*=0.42). This can be explained by the major differences between *S. cerevisiae* and *S. pombe*. As explained by [40], in *S. pombe*, DSB do not occur in most promoters and can occur in convergent regions. More precisely, in *S. cerevisiae*, 91% of divergent intergenic regions contain a DSB peak, while this number is only 70% in *S. pombe*. In *S. cerevisiae* the ratio between the number of DSB per kb in divergent versus convergent regions is around 14, while it is only 3 in *S. pombe*. Both these observations are in contradiction with our model, and that explains its poor performance for this species.

#### Comparison between SPoRE and other predictive tools

Several tools, based on nucleotide sequence analysis (considering k-mers, for *k*≥2) have been proposed [30-32] as predictors of recombination or DSB hotspots.

We compared to the most recent one, iRSpot-PseDNC [30], which improved above the others. In [30], the authors compared their predictions of DSB sites against 452 hotspots on chromosome IV extracted from the same Spo11 experimental data [9] that we compared to. They found that their program predicts as hot 347 of these hotspots, corresponding to a true positive rate of 77% [30]. When applying the same test to our model, we predicted as hot 361 of these 452 hotspots, corresponding to a true positive rate of 80%. However, to perform a proper benchmark, negative instances (coldspots) should be included in the test set, so that the false positive rate can also be measured. We therefore enlarged the dataset by adding 452 randomly chosen coldspots in the same experimental data and on chromosome IV (see Methods). On this symmetric test set, the overall success rate of iRSpot-PseDNC falls to 54% against 85% for our model (see Methods), compared to an expected 50% for a random prediction. This is due to the fact that iRSpot-PseDNC detects 309 false positives (false positive rate is 68%) while we only detect 43 of them (false positive rate is 10%). This shows that iRSpot-PseDNC is little better than random in detecting DSB hotspots. It should be noted that comparison is realized on hotspot sites localization but that no prediction on protein density is made by iRSpot-PseDNC, contrary to SPoRE, where estimations of density can be directly inferred from the modeling curve.

We also extended this benchmark over the whole *S. cerevisiae* genome by considering all the 3600 hotspots discovered in [9], together with 3600 randomly chosen coldspots. The accuracy of SPoRE in that case is 84% (close to 85% for chromosome IV). Its predictive performance can also be measured with a ROC curve by varying the density threshold, in which case the area under the curve is 0.90 (see Additional file 1: Figure S11). iRSpot-PseDNC success rate on properly identifying hotspots and coldspots is 55% (comparable to the 54% obtained on chromosome IV; due to the nature of iRSpot-PseDNC output, no ROC curve can be produced).

A second test was realized on the same dataset used in [30] to compare iRSpot-PseDNC to IDQD [31]. This dataset, defined in [31], is composed of 490 hot ORFs and 591 cold ORFs, where the hot ORFs describe a set of recombination hotspots. Notice that a recombination hotspot is expected to be located close to a DSB site but not the vice versa, and that SPoRE cannot be directly used for predicting recombination hotspots since it was designed to predict DSB hotspots.

Hence, we decided to test how much the smoothed GC-content, which we used as a factor in SPoRE, contributes to the identification of recombination hotspots. By using only GC-content, we obtained an accuracy of 83% (see Methods), against the 80% reached by IDQD and the 85% reached by iRSpot-PseDNC (based on a 5-fold cross-validation of the SVM approach they implement). The conclusion is that even though iRSpot-PseDNC is based on the actual DNA content (taking dinucleotide frequency as its predictor), it appears that almost all the signal can, in fact, be recovered simply with the GC-content in a window.

## Conclusions

We explored the hypothesis that genomic signals allow us to predict DNA double-strand breaks and the formation of the loops (their position and length) in the 3D chromosomal structure during meiosis. Our aim here is not to study the dynamics of a protein localization process but rather to identify the genomic information that can be used to predict the 3D structure formation and quantify the importance of these predictive factors. SPoRE allows us to test whether genomic signals are good predictive variables or not, and to what extent, in the accumulation of axis proteins and DSBs along chromosomes.

However, it should be noted that this does not imply that the factors are the cause of DSBs and axis proteins positioning. For example, GC-content could be a consequence rather a cause of DSBs [41]. In both cases however, it is a useful factor for predicting DSB hotspots.

All genomic factors considered in the model are linear functions with the exception of a quadratic factor modeling the impact of GC content. New parameters can be easily added to the model for the evaluation of new genomic markers effects. The interest in this modeling approach comes from a straightforward biological interpretation of the parameters that helps to reason on plausible biological mechanisms forming protein accumulation.

### Orientation of genes and chromosomal axis formation

We have shown through a formal model that the distribution of the chromosomal axis proteins is encoded in gene organization along DNA. The orientation of the genes influences the formation of the loops within the 3D axial structure during meiosis and to reach an understanding of this 3D structure formation, this fact should be combined with the existence of a random process governing the binding of the axis proteins to DNA and with a pervasive transcriptomic activity inducing a repositioning of the proteins in specific sites along the genome. In this respect, SPoRE model could help to design appropriate genomic signatures for synthetic chromosomes that should form a functional synaptonemal complex structure.

### Modeling organisms other than yeast

SPoRE could be used to infer localization and density of axis proteins and DSBs sites at large scale for those yeast species for which whole genome experiments have not been made yet. Today, more than 40 yeast genomes have been completely sequenced and for many of these yeast species, meiosis either exists or can be induced. It might be interesting to apply SPoRE model to these species to check, through comparative genomics, whether syntenic region boundaries correspond to DSB hotspots or not across species, whether the genetic content of DSB hotspots and of their neighborhoods are conserved in different species and so on.

Axial chromosome structures formation has been experimentally observed across many sexually reproducing eukaryotic species, from fungi to vertebrates. In yeast, our model highlights that axial chromosome structures and DSB distribution are governed by a rather simple combination of genomic signals. For other organisms, the model might be expected to become more complex. For the mouse, for instance, other factors such as DNA binding sites targeted by axial proteins have been demonstrated to play an active role in DSB localization [42]. In this respect, SPoRE might be taken as a nutshell to add extra signals and reach appropriate descriptions of experimental data in other organisms, possibly multicellular ones. SPoRE software is provided to allow users for further development and testing of new genomic factors.

## Methods

### Visualization in a genome browser

To allow biologists to visualize easily SPoRE modeling curves, SPoRE provides its results in the WIG file format. They can be loaded in the UCSC genome browser (http://genome.ucsc.edu/), in the genome browser available at http://yeastgenome.org/ and in the IGV software (see Additional file 1: Figure S1) [43]. For the four yeast genomes that we analyzed, the corresponding wig files are available at http://www.lcqb.upmc.fr/SPoRE/. For convenience, we also provide the corresponding *S. cerevisiae* experimental data in the same format, to allow for easy comparison.

### Software availability

SPoRE program is provided to the users that would like to apply it to yeast species, others than those we already considered here, or modify it for other organisms. The “readme” file explains what are the parameters that should be set for other organisms. The software is available at http://www.lcqb.upmc.fr/SPoRE/.

### Annotation

The reference strain we used to validate SPoRE is *Saccharomyces cerevisiae* S288C. The gene annotations were retrieved from the *Saccharomyces* Genome Database (http://www.yeastgenome.org/), release 64. We included 4879 “verified” ORFs and 895 “uncharacterized” ORFs in our set of coding genes, but not “dubious” ORFs. We also considered transposons by taking the 89 features labeled “transposable element gene”, rRNAs (RDN37-1, RDN37-2, RDN5-1, RDN5-2, RDN5-3, RDN5-4, RDN5-5, and RDN5-6), and pseudogenes (21). For *Lachancea kluyveri* and *Kluyveromyces lactis*, genomes and annotations were downloaded from Genolevures (http://www.genolevures.org/). Only features named “CDS” were taken into account in our models. For *Schizosaccharomyces pombe*, genome and annotation were downloaded from PomBase (http://www.pombase.org/). We used features labeled “CDS”, representing exons, and merged them together to get intervals defining genes in our models.

### Protein density data used for SPoRE validation

We use protein density data along the genome from Spo11 immunoprecipitation/454 sequencing for DSB [9] and from ChIP-on-chip for Red1, Hop1 and Rec8 [24]. They were mapped on the *S. cerevisiae* S288C genome, even though strain SK1 was used in the experiments. Raw data were used for computing all correlations reported in Table 1. They were retrieved from supplementary data in [9] for Spo11, and from the GEO dataset GSE29860 for Red1/Hop1/Rec8.

### Smoothing

To smooth the curves, we use a kernel-based smoothing with a Gaussian kernel. We use the “density” function provided in R [44] for all our models, the Spo11 experimental data and the GC-content. We use the “ksmooth” R function for Red1 experimental data to take into account correctly the irregular spacing of the tiling array probes. When referring to *σ* nt smoothing, we mean that the Gaussian kernel we use has a standard deviation of *σ*.

For DSBs, we used *σ*=250 nt for both data and models. Notice that Spo11 experimental data have a nucleotide-level precision and that the smoothing we use takes into account the range in which Spo11 might cut DNA around hotspots. For axis proteins, we used *σ*=1000 nt for the Red1 experimental data, and *σ*=1500 nt for our models. The rationale behind the different values is that ChIP-on-chip experiments produce large fragments of DNA where proteins bind and, as a consequence, a large range of probes in the microarray detects them. The accumulation of probes does the equivalent of a smoothing, and because of this, we need to smooth the data less than in the model. The two parameters were adjusted so that the number of peaks detected on both smoothed curves is approximately the same (1558 for *S. cerevisiae* data, 1615 in SPoRE model). More precise experimental data might correspond to a different smoothing constant *σ* and the software allows for easy changes.

### Normalized density and experimental noise

Normalized density (*y* axis in Figure 3 and Additional file 1: Figure S6) is defined by translating and scaling the values in such a way that the first percentile maps to 1 and the 99th percentile maps to 99. This is a way to scale the data approximately between 0 and 100 without taking into account extreme values. In fact, these latter might be a consequence of the experimental noise. In Red1 model 4 (Table 1), noise was estimated from data by considering the 1st percentile *m* and the 99th percentile *M*, where *m*=2.169456 and *M*=7.622778 for *S. cerevisiae*. The ratio *M*/*m*=3.5 has been used to estimate the noise level in Figure S3E.

### Correlations between model and experimental curves

To estimate the local correlation between two curves, we considered a window of 50 kb in which we compute the correlation coefficient (Pearson or Spearman) between points of the two curves every 10 nt. Then we move the window by 10% of its size (ie. 5 kb) and repeat the computation until we reach the end of the chromosome. We repeat these operations for each chromosome, and finally, we take the average of all these correlation coefficients (from all windows from all chromosomes).

Global correlation is computed by considering the complete genome at once (all points every 10 nt), instead of a sliding window. It provides a single correlation coefficient.

### Peak predictions and their evaluation

High-density spots for both axis proteins and DSBs are computed as the peaks of the corresponding smoothed curves. They are defined as local maxima that are at least *ε*=1 normalized density unit (see “Normalized density and experimental noise”) above the surrounding local minima.

To evaluate high-density spot predictions versus experimental hotspots, we used two standard measures, sensitivity and Positive Predicted Value (PPV). Namely, for each peak in the experimental curve at position *x*, we look for a peak in the model lying in the interval [*x*−*Δ*,*x*+*Δ*]. If there is such a peak then we count it as a true positive. Sensitivity is defined as the fraction of true positives over the number of real peaks. Positive Predictive Value is defined symmetrically to sensitivity, by reversing real and predicted peaks. It is the fraction of real peaks over the number of predicted peaks.

### Random models for axis proteins and DSBs sites

In order to test whether sensitivity and PPV values scored by SPoRE for axis proteins and DSB spots predictions are not the result of chance, we generated 1000 random models for the two kinds of loci. For axis proteins, the models were generated by randomly selecting 1615 positions along the whole *S. cerevisiae* genome, that is, the same number of peaks as in SPoRE model 3 in Table 1. For DSB spots, the models were generated by randomly selecting 4242 positions in *S. cerevisiae* intergenic regions, that is, the same number of peaks as in SPoRE model 6 in Table 1. We explicitly considered intergenic regions because it is already known that DSB spots occur there. We wished to test whether our predictions are closer to real axis proteins or DSB spots than a random choice. After generating the random positions, we evaluated the position against experimental peaks by using the same method employed for SPoRE (see above).

### Intergenic region lengths

SPoRE model for DSBs uses intergenic regions lengths as a contributing weight. Precisely, given an input genome, we compute the distribution of its intergenic region lengths and set the threshold IRL_*max*_=*μ*+*σ*, where *μ* and *σ* are average and standard deviation of the distribution. For *S. cerevisiae*, this value is 1202 nt (the first analysis of these regions in *S. cerevisiae* dates back to [45]). For intergenic regions that are “too large”, that is >*μ*+*σ*, we set the weight to IRL_*max*_, that is, the weight stops growing after the threshold.

### GC content

When taking into account GC content in our model, we use a kernel-based smoothing of the GC distribution on all nucleotides along the genome (both genic and intergenic), obtained from a Gaussian kernel with standard deviation 1 kb. Then we define all GC-based values with the smoothed GC curve: *μ*_GC_, *σ*_GC_ and gc(*p*_*g*_) (see above). All along the genome, we assume the presence of a minimal amount of GC content expressed by the threshold GC_min_=*μ*_GC_−3*σ*_GC_.

### Gene projections

Plots in Additional file 1: Figure S2, Additional file 1: Figure S3 and Additional file 1: Figure S4, were created by first smoothing the experimental data, then summing Red1/Spo11 smoothed curves after centering them on reference positions (gene 5’-end, gene 3’-end, intergenic region centers). The smoothing Gaussian kernel standard deviation used is *σ*=20 nt, except for Additional file 1: Figure S4B and Additional file 1: Figure S4D where we used respectively *σ*=15 nt and *σ*=5 nt. When smoothing Red1 data, some values are missing because sometimes probes are too far from each other, so the curve cannot be computed by using the Gaussian kernel between them. To avoid this problem, we removed the intergenic regions with such holes in the gene projection plots. More precisely, we removed 1 intergenic region out of 371 from the red curve in Additional file 1: Figure S4A and 16 intergenic regions out of 381 from the yellow curve in Additional file 1: Figure S4A and Additional file 1: Figure S4B.

### Promoters and Transcription Factor Binding Sites (TFBS)

SPoRE can model DSBs either by approximating the position of promoter regions proportionally to the length of the associated intergenic region (see DSB model description above), or by exploiting knowledge of TFBS when available. For the latter, given a gene, it considers the set of its TFBS and computes the average of their positions as the reference position to set the weight of the SPoRE model. In case a gene has no known TFBS, then SPoRE models its promoter location based on the length of its intergenic region. For *S. cerevisiae*, we used TFSB positions indicated in the Yeast Promoter Atlas [36] repository, available at http://ypa.csbb.ntu.edu.tw.

### Comparison with iRSpot-PseDNC on DSB data

Comparison between SPoRE DSB model and iRSpot-PseDNC [30] was realized on the dataset of 452 experimentally annotated [9] recombination hotspots for the *S. cerevisiae* chromosome IV. This set, originally used to evaluate iRSpot-PseDNC in [30], has been extended with 452 coldspots that we extracted from the same experiment [9]. This extension was done in order to test both systems for false positives. More precisely, for each hotspot in the dataset, we randomly selected a fragment of DNA on chromosome IV with the same length as the hotspot, but without any experimentally detected DSB, and verified that these fragments do not overlap each other. (Notice that 17% of the *S. cerevisiae* genome is made of regions that are larger than 242 nt, that is the average size of a hotspot, and that contain no peak. We have randomly selected coldspots within these regions). Hence, we obtained a set of coldspots with the same number of sequences and the same length distribution as the set of hotspots. We then tested iRSpot-PseDNC online by providing the server with the DNA sequences in the dataset (the file is available at http://www.lcqb.upmc.fr/SPoRE/). To test our model, we simply predicted as a hotspot any fragment on which the average of our curve is higher than the average over the whole genome.

To generate Additional file 1: Figure S11, we considered hotspots and coldspots over the whole *S. cerevisiae* genome. We used the same process as explained above for choosing coldspots, with the only differences that, first, we repeated the process for each of the 16 chromosomes, and, second, that we allowed for at most 1 read to be present in a coldspot (requiring 0 reads is too stringent on some parts of the genome). We then used the average of our curve over the hotspots and coldspots as a predictor, and varied the threshold to produce the ROC curve (instead of setting it to the mean as above).

A second dataset was used for comparison with iRSpot-PseDNC and IDQD [31]. It is defined in [31] and it is downloadable as SI of [30]. This set is defined by ORFs, but since SPoRE uses information about intergenic regions instead, we benchmarked SPoRE on this dataset by predicting hotspots on the intergenic regions lying before the gene start. Namely, we compared the average of our modelling curve in this region to its mean *μ* and standard deviation *σ* by predicting hotspots when the average of the curve is ≥*μ*+*σ*. When the GC-content curve has been tested as a predictor of recombination hotspots in this dataset, formally, we compared the maximum of the smoothed GC-content curve in the gene and intergenic region preceding it to *μ*_GC_+*σ*_GC_, where *μ*_GC_,*σ*_GC_ are the mean and the standard deviation of the GC-content curve on the full genome. Notice that a much simpler model could replace this GC-curve. In fact, we could just consider a 4kb window centered at the start of a gene, compute its GC-content, and obtain identical accuracy.

## Additional file

Additional file 1 **Supplementary Material.**
