# Supplementary material for: SPoRE: a mathematical model to predict double strand breaks and axis protein sites in meiosis
Source: BMC Bioinformatics. 2014 Dec 11;15(1):391. doi: 10.1186/s12859-014-0391-1 (PMC4268827; doi:10.1186/s12859-014-0391-1)
Supplement: Supplementary file 1 — Supplementary Material. [file 12859_2014_391_MOESM1_ESM.pdf]

Supplementary Material

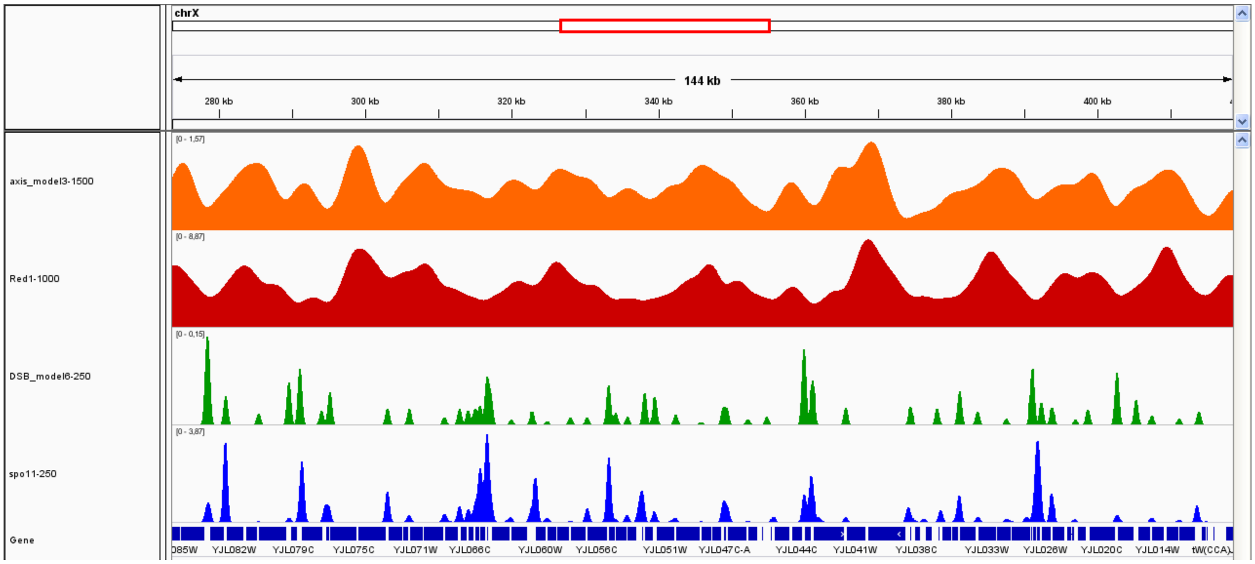

Figure S1. Screenshot of IGV [44]. Screenshot obtained after loading SPoRE modeling curve for axis proteins (orange) and DSBs (green) and the corresponding experimental data for Red1 (red) and Spo11 (blue). Gene location is reported on the bottom of the page (dark blue rectangles).

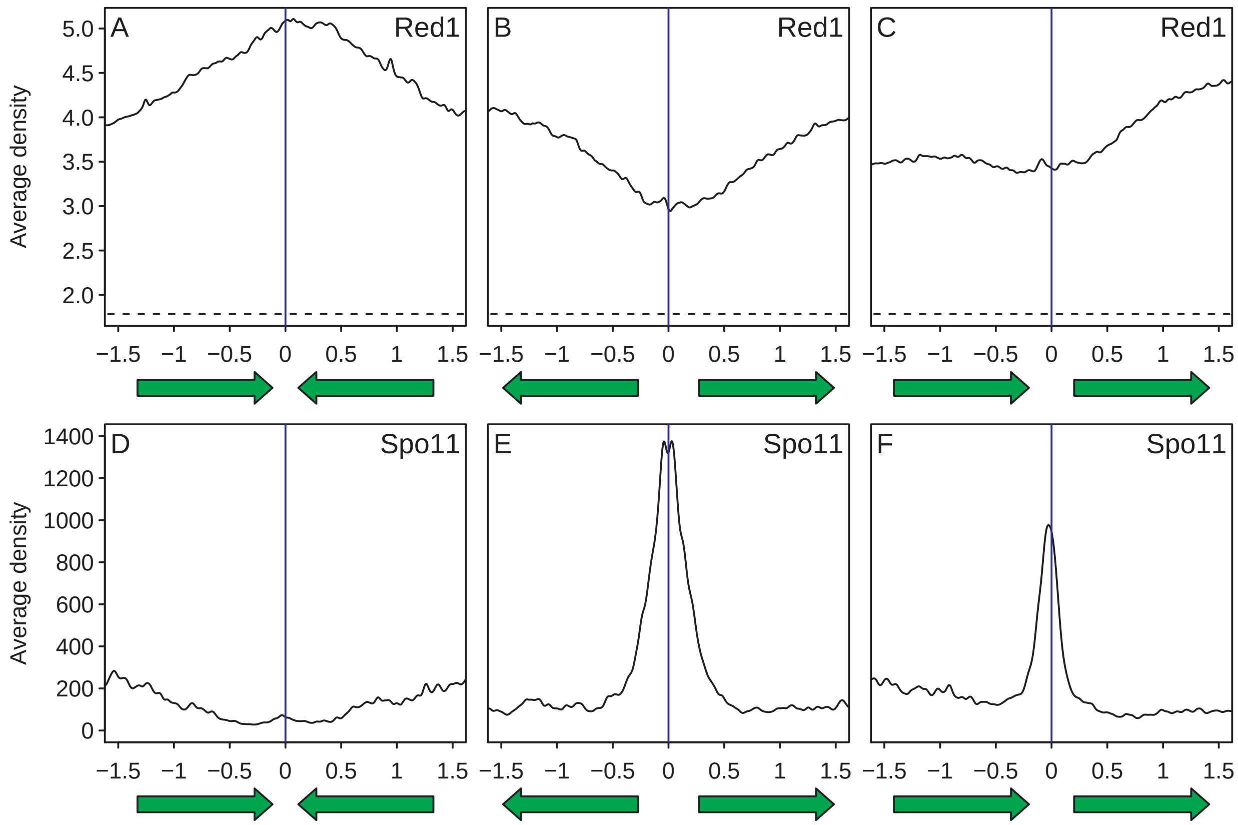

Figure S2. Proteins density in convergent and divergent intergenic regions of the *S. cerevisiae* genome. Average Red1 density [24] is computed in a 3 kb window around intergenic region centers, for regions with convergent genes (A), divergent genes (B), or tandem genes (C). The scale is defined as microarray intensity. The dotted line shows the "base noise level" corresponding to the first decile (1.78) in the intensity distribution, considered as "no or low signal" in [24]. Compare the curves in ABC with the corresponding curves for Hop1 and Rec8 axis proteins in Additional file 1: Figure S10.

Plots D, E and F show the same distributions as in plots A, B and C, but for Spo11. They are based on experimental data, measured in reads per kb, reported in [9].

Genes drawn below plots show the median gene length (1212 nt) and the median intergenic region length for the type of region shown (231, 545, and 410 nt for convergent, divergent, and tandem regions, respectively). When comparing the densities at the center of intergenic regions (vertical blue lines) between different intergenic region types, we find that all the differences are statistically significant, with all p-values lower than $10e^{-5}$ (using Welch two sample t-tests).

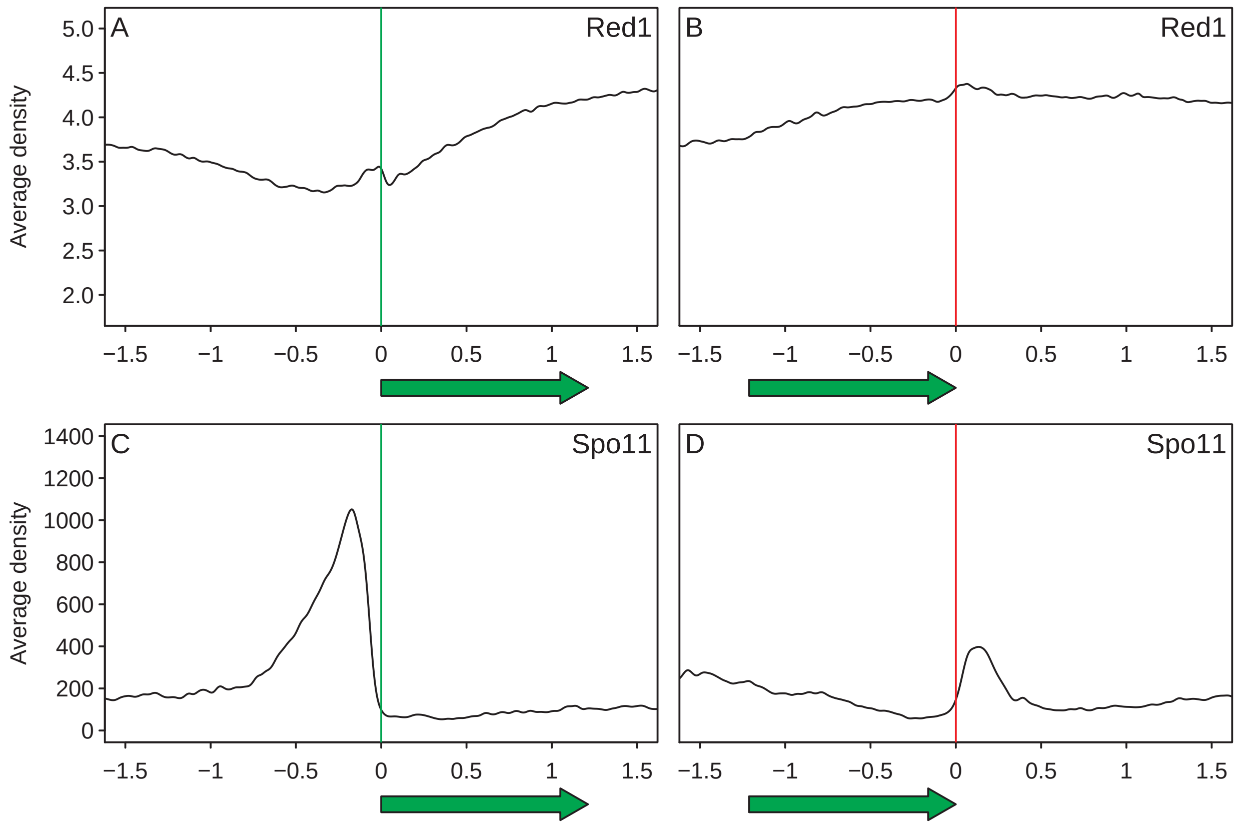

Figure S3. Average Red1 density [24] in a 3 kb window centered at gene 5'-ends (A) and gene 3'-ends (B). The zero is respectively the gene start and stop. Plot C shows the average Spo11 density at gene 5'-ends observed [9], while figure D shows it at gene 3'-ends.

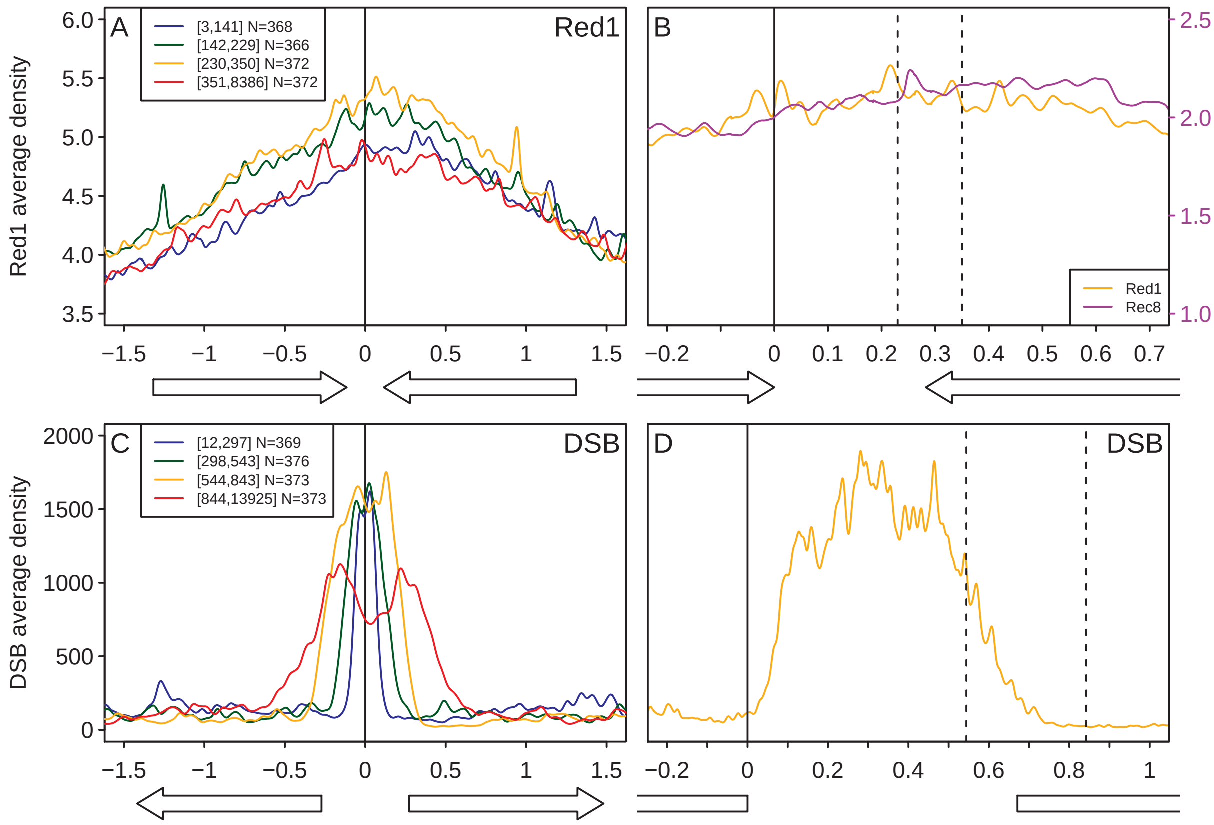

Figure S4. Proteins density in intergenic regions for different intergenic region lengths. (A) Detailed analysis of the Red1 density curve in Additional file 1: Figure S2A. The full set of intergenic regions delimited by convergent genes, is studied here by considering quartiles defined on the intergenic region lengths distribution. The four average Red1 density curves [24] are plot on a 3 kb window around intergenic region centers for each quartile, and are distinguished by different colors. The figure legend reports the interval $[x, y]$ of intergenic region lengths associated to each quartile and the number N of its intergenic regions. The vertical axis corresponds to the Red1 average density scale, measured with microarray intensity values given in [24]. (B) Detailed view of the third quartile data in A (yellow curve). The intergenic regions have been centered on the left gene ends (vertical solid line at 0 kb). The two vertical dotted lines show the interval $[230, 352]$ in which right genes end. Rec8 density is shown for comparison (purple curve). The vertical axis on the right indexes Rec8 average density. (C,D) The same analysis as in A and B is realized for Spo11 [9] on divergent intergenic regions. The vertical axis measures the number of reads per kb.

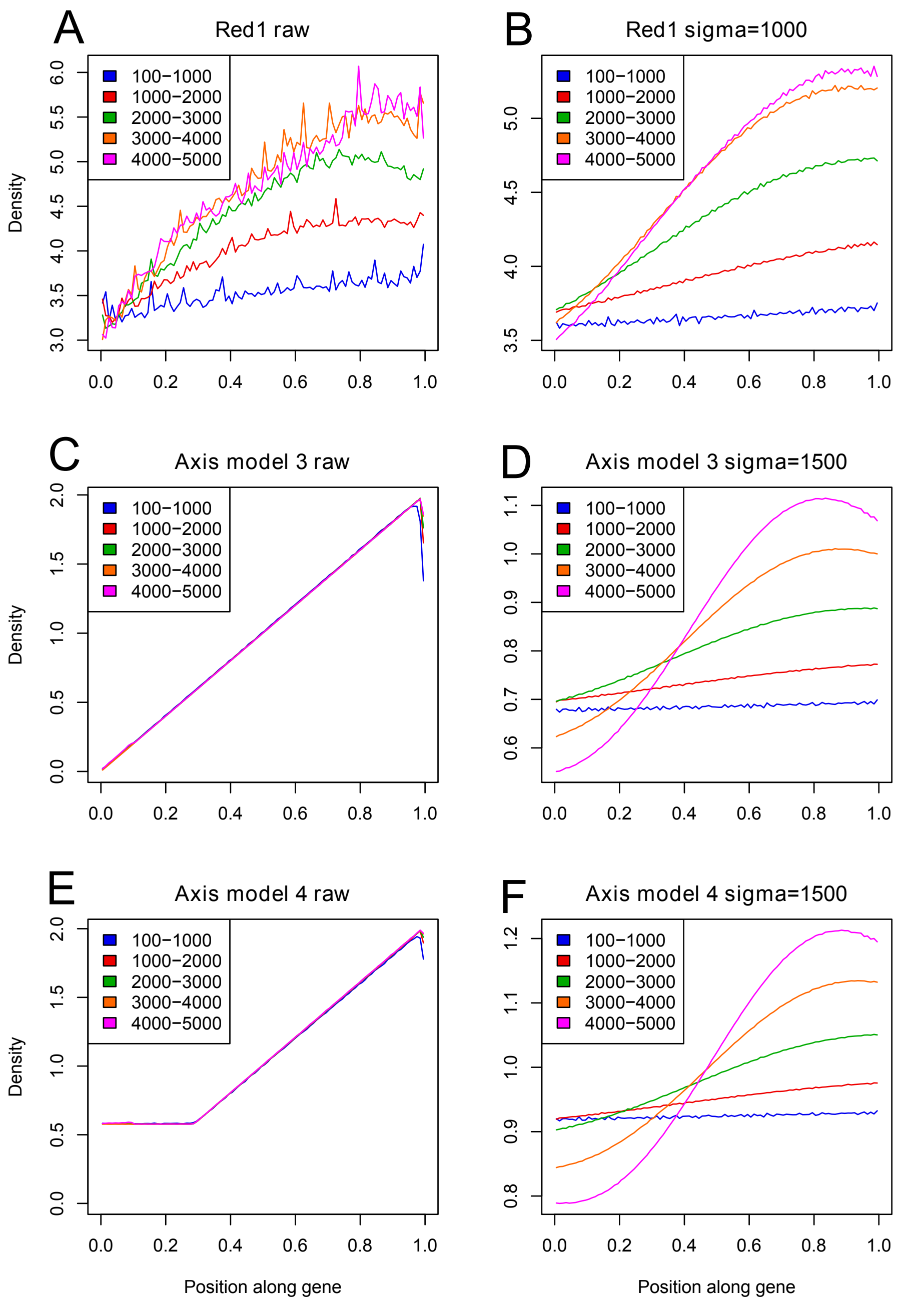

Figure S5. (A) Experimental curves describing the accumulation of Red1 proteins in *S. cerevisiae* [24], along sets of genes of comparable length. Five different curves are plot, corresponding to gene lengths in the intervals $[100, 1000]$, $(1000, 2000]$, $(2000, 3000]$, $(3000, 4000]$ and $(4000, 5000]$. Gene lengths are normalised over the interval $[0, 1]$. (B) The experimental distribution curve in A has been smoothed (with a smoothing coefficient $\sigma = 1000nt$) along the entire chromosomes and the resulting smoothed curve corresponding to gene regions is plot again for sharper visualisation. (C) Modelling curve used to approximate experimental data in SPoRE. (D) The modelling distribution curve in C has been smoothed (with a smoothing coefficient of $\sigma = 1500nt$) along the entire chromosomes and the resulting smoothed curve corresponding to gene regions is plot again for sharper visualisation. (E) As in C, but including background noise (model not included in Table 1; see text). Noise level is computed from experimental data in [24]. (F) As in D, but based on the modelling distribution curve in E.

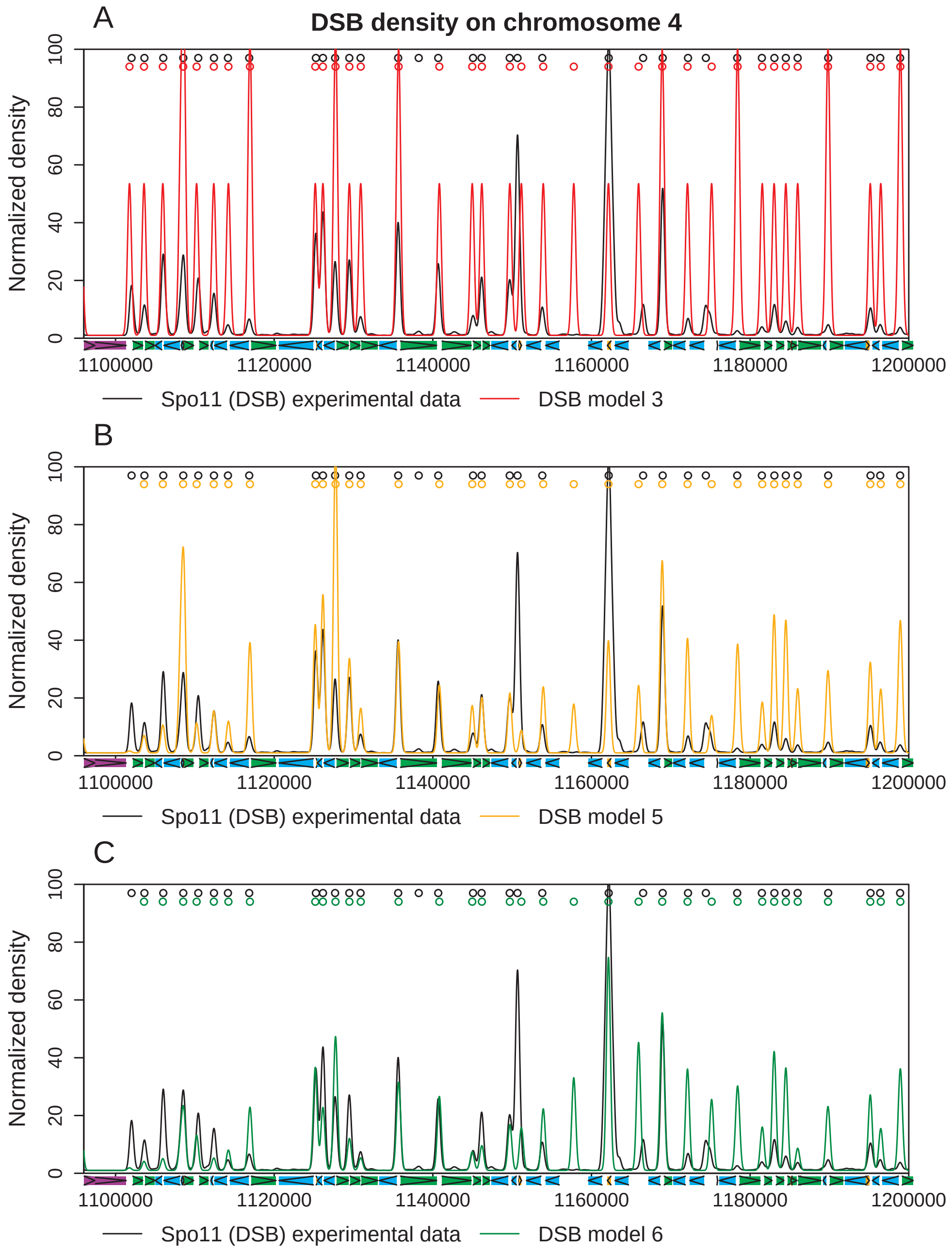

Figure S6. DSBs models compared to experimental data on a zoomed region of chromosome 4. Different models listed in Table 1 (red, blue and green curves) were compared to experimental data [9] on a 100 kb region of chromosome 4. Genes along the region are highlighted by different colors: forward genes in green, backward genes in blue, transposons in purple and "dubious" genes (not considered as genes in our models) in orange.

(A) Model 3 is characterised by constant weights (red curve).

(B) Model 5 is weighted with GC-content (blue curve).

(C) Model 6 (SPoRE) is weighted with GC-content and intergenic region lengths (green curve). See Figure 5 for a heatmap along the whole genome.

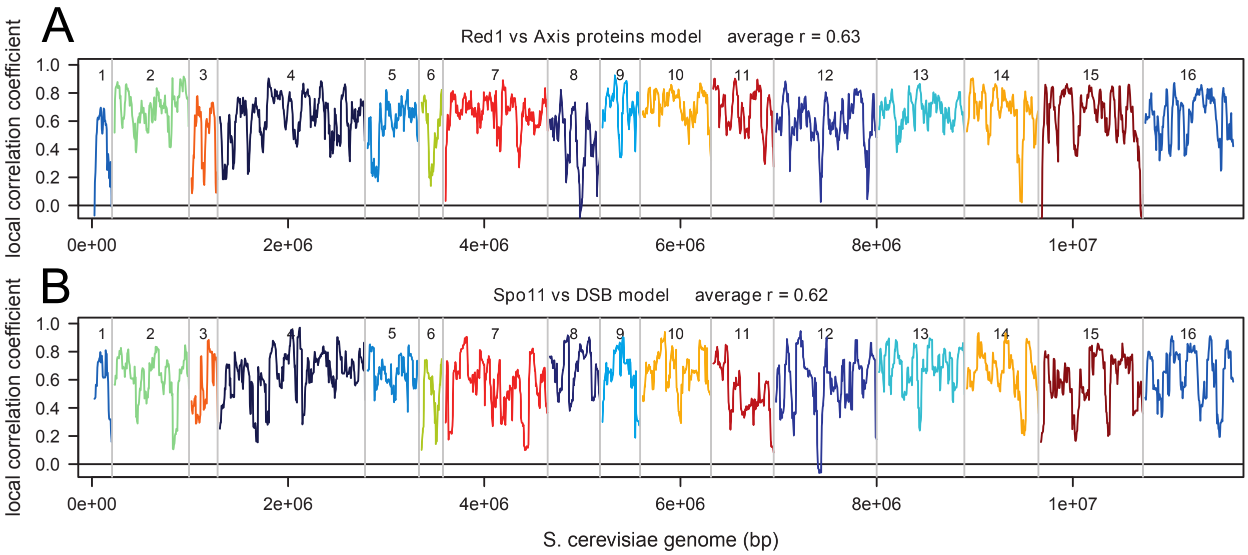

Figure S7. (A) Local correlation, colored by chromosome, between the Red1 experimental data [24] and the SPoRE axis proteins model, along the whole genome. (B) As in A, with Spo11 experimental data [9] and the SPoRE DSBs model.

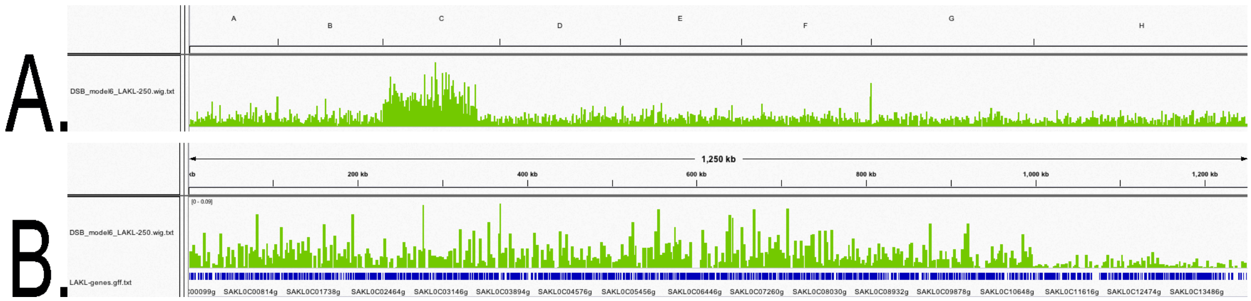

Figure S8. Predicted landscape of DSB distribution in *L. kluyveri*. (A) IGV screenshot of SPoRE DSB model of the full *L. kluyveri* genome. (B) IGV screenshot of SPoRE DSB model on *L. kluyveri* chromosome C. Notice that the left chromosomal arm displays a high DSB density compared to the right arm. The centromere is located approximately at position 1000kb.

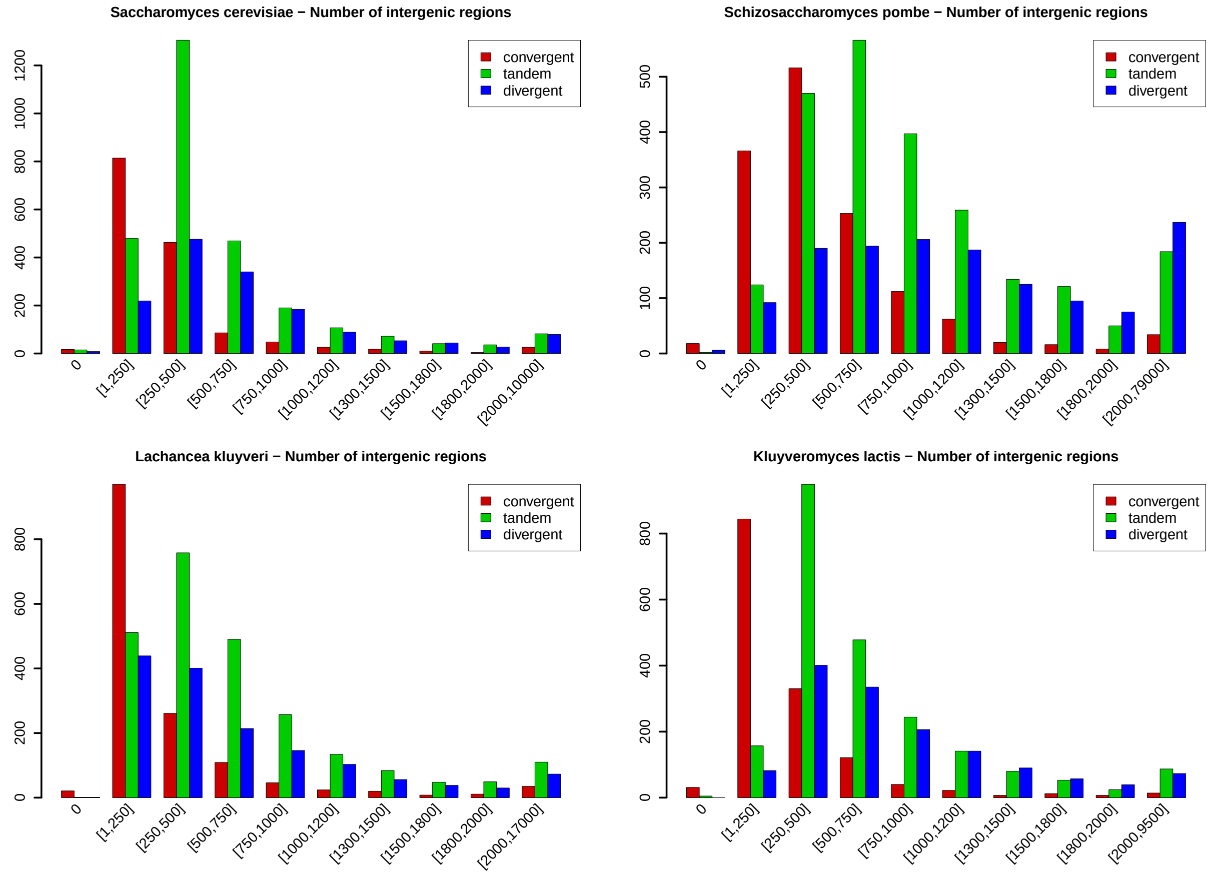

Figure S9. Distribution of intergenic regions lengths in four yeast species: *S. cerevisiae* (top left), *S. pombe* (top right), *L. kluyveri* (bottom left), *K. lactis* (bottom right). Intergenic regions are organised in convergent (red), divergent (blue) and tandem (green) regions.

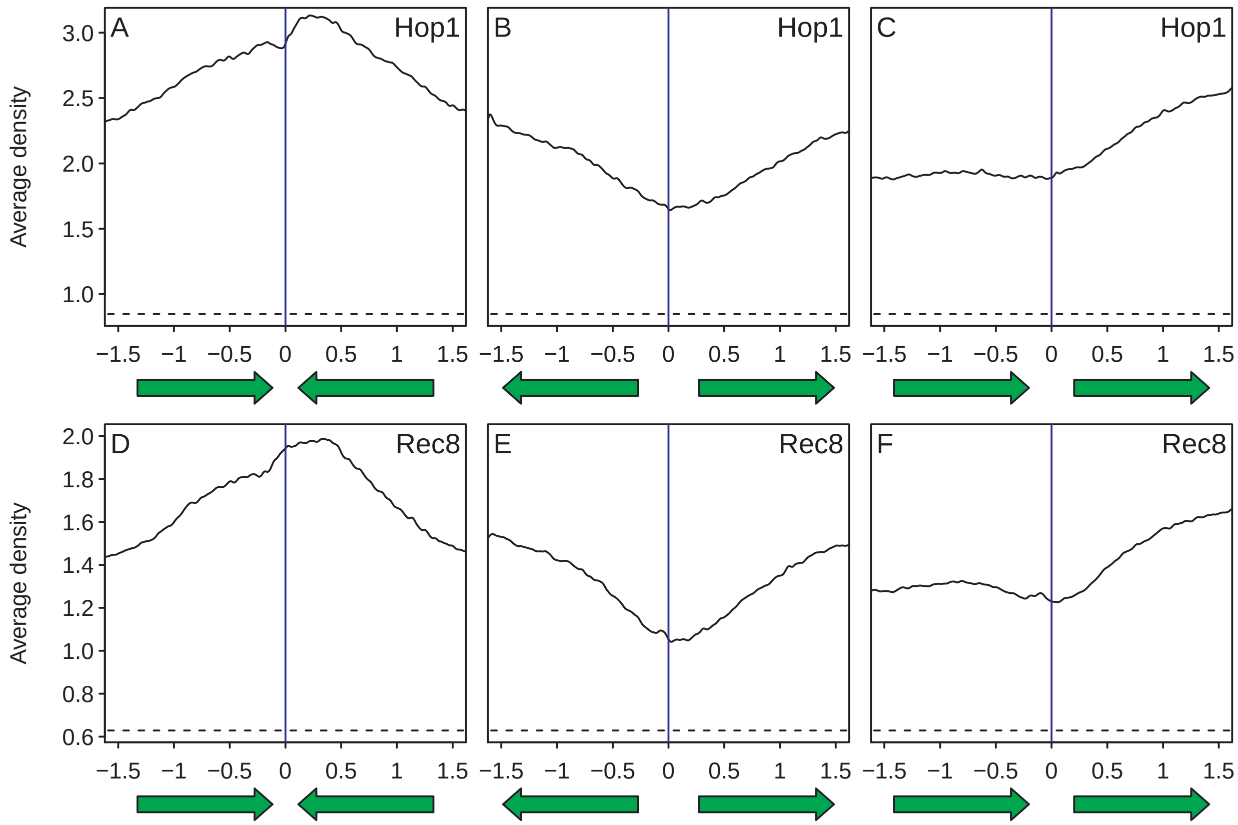

Figure S10. Proteins density in convergent and divergent intergenic regions of the *S. cerevisiae* genome. Average Hop1 density [24] is computed in a 3 kb window around intergenic region centers, for regions with convergent genes (A), divergent genes (B), or tandem genes (C). The scale is defined as microarray intensity. The dotted line shows the "base noise level" corresponding to the first decile (0.85) in the intensity distribution, considered as "no or low signal" in [24]. Plots D, E and F show the same distribution for the Rec8 protein, based on [24] experimental data. The base noise level for Rec8 is 0.63. Genes drawn below plots show the median gene length (1212 nt) and the median intergenic region length for the type of region shown (231, 545, and 410 nt for convergent, divergent, and tandem regions, respectively). Compare to the Red1 distribution plots in Additional file 1: Figure S2ABC.

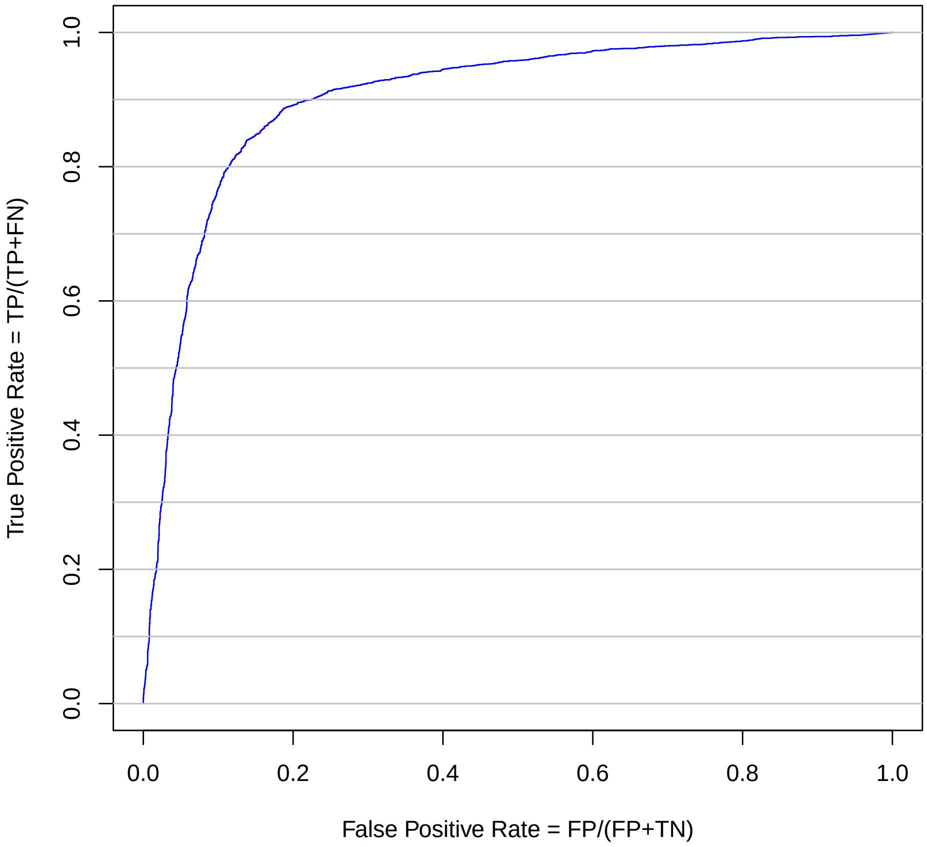

Figure S11. ROC curve showing SPoRE prediction performance on DSB hotspots over the *S. cerevisiae* complete genome. A total of 3600 hotspots from [9] were considered, in addition to 3600 coldspots randomly chosen from the same data. The area under the curve is 0.90.
